# Pathways to food insecurity: Migration, hukou and COVID‐19 in Nanjing, China

**DOI:** 10.1002/psp.2640

**Published:** 2022-12-30

**Authors:** Fei Xu, Jonathan Crush, Taiyang Zhong

**Affiliations:** ^1^ School of Geography and Ocean Sciences Nanjing University Nanjing China; ^2^ Balsillie School of International Affairs Waterloo Ontario Canada; ^3^ University of the Western Cape Cape Town South Africa

**Keywords:** food security, Hukou, insufficient food quantity, nonlocal households

## Abstract

The COVID‐19 pandemic has issued significant challenges to food systems and the food security of migrants in cities. In China, there have been no studies to date focusing on the food security of migrants during the pandemic. To fill this gap, an online questionnaire survey of food security in Nanjing City, China, was conducted in March 2020. This paper situates the research findings in the general literature on the general migrant experience during the pandemic under COVID and the specifics of the Chinese policy of hukou. Using multiple linear regression and ordered logistic regression, the paper examines the impact of migration status on food security during the pandemic. The paper finds that during the COVID‐19 outbreak in 2020, households without local Nanjing hukou were more food insecure than those with Nanjing hukou. The differences related more to the absolute quantity of food intake, rather than reduction in food quality or in levels of anxiety over food access. Migrants in China and elsewhere during COVID‐19 experienced three pathways to food insecurity—an income gap, an accessibility gap, and a benefits gap. This conceptual framework is used to structure the discussion and interpretation of survey findings and also has wider potential applicability.

## 1 INTRODUCTION

The COVID‐19 pandemic has had a significant negative impact on the global food system, disrupting international and national food supply chains, increasing food prices, and reducing consumer access to affordable foods. One consequence has been a sharp increase in the prevalence of food insecurity in many countries. In 2020, as many as 800 million people in the world faced hunger, an increase of 161 million from 2019. A total of 2.37 billion people were without access to adequate food in 2020 (FAO, 2021). After remaining virtually unchanged from 2014 to 2019, the PoU (Prevalence of Undernourishment) increased from 8.4% to around 9.9% between 2019 and 2020 (FAO, 2021). To control the pandemic, many governments implemented mitigation measures including lockdowns, stay‐at‐home orders, mobility restrictions, and closure of public events and spaces (Hale et al., 2021). While these measures helped reduce the spread and number of fatalities from COVID‐19, they also posed a significant threat to the food security of urban populations across the Global South (Crush & Si, 2020).

The pandemic has been particularly severe on the food security of the urban poor and marginalized, including many international and internal migrant workers (Crush et al., 2021). McAuliffe et al. (2022) describe COVID‐19 as the ‘great disrupter’ for migrant workers. Many of the world's 280 million international and 750 million internal rural‐urban migrants are precariously employed in labour‐intensive, low‐paid (often informal), 3D (dirty, dangerous, demeaning) jobs with little employment security and limited access to social protection programmes. Although these conditions and vulnerabilities pre‐date the pandemic, their consequences have been seriously exacerbated by COVID‐19 (Fassani & Mazza, 2020; de Haan, 2020; Rajan, 2020; Suhardiman et al., 2021). Migrants were laid off in large numbers as businesses shut down and reduced their employment rolls in the early months of the pandemic. Those who retained their jobs were particularly vulnerable to infection in unregulated and over‐crowded workplaces without adequate PPE (Landry et al., 2021; Reid et al., 2021). Migrant workers were often quarantined in over‐crowded accommodation, further increasing their vulnerability to infection and death (Alahmad et al., 2020; Yee et al., 2021).

In many countries, there was a ‘remittances shock’ as transfers to family at home declined (Caruso et al., 2021; Takenaka et al., 2020; Withers et al. 2022). Internal urban‐rural remittance flows also declined (Rajan & Bhagat, 2022). As the IMF has noted, ‘sharp output contraction, together with travel restrictions in major migrant hosting economies, jeopardized migrants' employment countries and income opportunities and brought into question remittances' ability to smooth consumption in home countries’ (Kpodar et al., 2021). The pandemic also imposed major constraints on international mobility, trapping migrants in destination countries as road, rail and air transportation halted and borders were closed to all but essential workers (Ullah et al., 2021). By contrast, internal migrants facing unemployment, food insecurity and COVID‐19 infection began moving en masse from the cities back to their rural homes in many countries (Irudaya Rajan et al., 2020; Mukhra et al., 2020)

Although there is a growing literature on the experiences of migrants during successive waves of the pandemic, the impact on migrant food security is underexplored (Crush et al., 2021; Ramachandran et al., 2022; Sharma, 2020). In China, studies have shown the negative impact of COVID‐19 on migrant employment (Che et al., 2020), remittances (Zhang et al., 2021) and access to social protection (He et al., 2022), but have not specifically focused on the food security of migrants. The Zero‐Covid policy meant that the first wave of the epidemic was relatively short‐lived, although emerging research suggests that there was a general increase in food insecurity in Chinese cities (Dou et al., 2021; Zhan & Chen, 2021). In this paper, we aim to contribute to a better understanding of the complex relationship between COVID‐19, migration, and food security through an analysis of the impact of the pandemic on rural‐urban migrants in China.

The paper also augments the literature on the migration and food security nexus during COVID‐19 in three main ways. First, the paper builds on the growing body of evidence on the impact of COVID‐19 on food system disruption and resilience with their associated challenges which, in China, included food price increases (Ruan et al., 2021; Yu et al., 2020), changes in household food purchasing behaviour (J. Li et al., 2020, S. Li et al., 2022; Yue et al., 2021), and the dramatic growth of online food purchasing (Dai & Qi, 2020; Gao et al., 2020; Liang et al., 2022; Lu et al., 2021). Second, the paper adds to a small number of case studies of the impact of COVID‐19 on household food consumption and food security in Chinese cities to expand our knowledge of the food security experience of the large Chinese urban migrant population during the pandemic (Cui et al., 2021; S. Li et al., 2022; Zhang et al., 2020; Zhao et al., 2020). Third, the paper develops a theoretical framework with broader applicability which highlights the potential pathways towards food insecurity confronting migrants during the pandemic.

The paper is organized as follows. The next section of the paper provides a contextual overview of internal migration and the Chinese hukou (household registration) system, the current state of knowledge of the impact of COVID‐19 on migrants in cities, and a theoretical framework for conceptualizing the connections between migration and household food security during COVID‐19. The following section of the paper describes the research methodology involved in collecting household‐level data during the pandemic in the case study city of Nanjing as well as the food security indicators used in the data set. The paper then analyses the survey data using descriptive statistics and regression modelling, before concluding with recommendations for additional research.

## 2 INTERNAL MIGRATION, HUKOU AND COVID‐19

### 2.1 Internal migrants and the hukou system

In recent decades China has undergone a major transformation from a predominantly rural to a majority urban country (Fan, 2007; Lu & Xia, 2016; Tang, 2012). The proportion of China's population that is urbanized increased from 20% in 1980 to 64% in 2020 (National Bureau of Statistics, 2021a; State Council, 2021). Urbanization in China is closely related to the longstanding Chinese policy of hukou or household registration (K. W. Chan & Wei, 2019; K. Chan & Yang, 2020). There are two main types of urban resident: the population with local hukou in cities and those with hukou in other, predominantly rural, areas. The latter are often referred to as rural‐urban migrants or the ‘floating population’ (Liang et al., 2014; Shen et al., 2022). Their number increased from 155 million in 2010 to 376 million in 2020 (National Bureau of Statistics, 2021a). Migrants made up 16.5% of the total population in 2010 and 26.6% in 2020. Most of China's floating population is concentrated in the country's mega‐cities of Shanghai, Beijing, Guangzhou, and Shenzhen. About 44% of the population in cities with over five million people are migrants (K. Chan, 2019, 2021). Most migrants are employed in export‐oriented manufacturing, construction, sales, domestic work, and food services. Migrants often work long hours, have little job security and few benefits. H. Cheng et al. (2020), for example, report a significant wage differential between migrant and urban workers, largely attributed to the individual characteristics and human capital levels of rural versus urban dwellers

Since the 1950s, the hukou system has acted as an important determinant of the pace and spatiality of rural‐urban migration and the prospects for permanent urban residence. All Chinese people are registered at birth at the local police station in the prefecture in which they are born (Luo et al., 2019). Each household has a hukou registration document which contains information on the household head, the household members, and home address. Members of households with rural hukou are not stopped from migrating to the cities to live, work or study but are categorized as nonlocal or floating (National Bureau of Statistics, 2021b). Hukou is thus both an information system of prefecture level registration and an identity label that distinguishes between local and nonlocal residents and their entitlements.

In 2014, China launched a major initiative to reform the hukou system by promoting the conversion of rural to urban hukou by migrant households (K. Chan, 2019; Government of China, 2014; B. Li et al., 2016; State Council, 2014). The conversion programme incentivizes rural‐urban migrants to move to smaller cities where they can access a broader range of opportunities and benefits (Raimondo, 2019; Yang & Guo, 2018). Cities with populations between one and three million dropped all restrictions on household registration. Cities of three to five million were scheduled to relax restrictions on new migrants and remove limits on key population groups, including university graduates. By 2020, 100 million migrants had accessed the new policy (K. Chan, 2021). However, 13 cities, including Nanjing, are not scheduled for a relaxation of hukou restrictions. Although conversion is an incentive for many, not all migrants wish to convert to urban hukou (C. Chen & Fan, 2016; Hao & Tang, 2015; Tang & Hao, 2018).

### 2.2 Existing studies on the impact of COVID‐19 on migrant food security

Holdaway (2015) draws attention to the pre‐pandemic food security implications of nonlocal hukou status for migrants in cities, noting that they are ‘a potentially vulnerable population in the urban context because their low incomes, long working hours and poor housing conditions limit their choice in terms of what they eat and how it is prepared’. The links between food consumption, nutritional status, and health outcomes of migrants in the city have been explored in several studies. Sun (2021) and Sun and Li (2021), for example, use national survey data for over 7500 migrant households to show that urbanicity (the degree of urban infrastructure where migrants live) has a significant impact on food intake and health. Sun et al. (2021) also found a significant gender effect on energy intake and its share from protein amongst migrants. Z. Cheng (2021) shows that dietary quality is positively associated with migrants' level of education. Comparative studies include Liu et al. (2022) on variations in children's nutritional status between rural hukou households in cities and the countryside. Liao (2018) shows that in Shanghai migrant households actually have more diverse and nutritious diets than local households. Other studies have compared patterns of food consumption by migrants and local urban households and attributed differences to the hukou system (B. Chen et al., 2015; Han et al., 2019; J. Wang et al., 2021).

In early 2020, strategies to control the spread of COVID‐19 had a major impact on the everyday lives and food consumption patterns of residents of Chinese cities (Zha et al., 2022; Zhong, Crush, et al., 2021). While Wuhan was the only city to experience a complete residential and workplace lockdown, many cities implemented policies that curtailed the mobility of the population and its access to income earning opportunities, to educational institutions, and to normal food sources such as wet markets and supermarkets. Evidence is beginning to emerge that migrants in cities were especially affected. Che et al. (2020), for example, estimate that at least 30–50 million migrants lost their jobs by late March 2020. He et al. (2022) note that migrants were hard hit by layoffs in labour‐intensive, export‐oriented industries, Zhang et al. (2021) found that 70% of migrant workers lost part of their wage income during the pandemic lockdown period and those working in small and medium enterprises were most affected. About 50% of remittance‐receiving households in rural areas were adversely affected by decliningremittances with an average decline of 45%. These pandemic‐related impacts on the livelihoods of migrants would, in theory, have had spin‐off effects on their food security in the cities.

### 2.3 Conceptualizing migrant pathways to food insecurity

Research on the impacts of COVID‐19 has increasingly focused on the pre‐pandemic conditions that rendered some groups more vulnerable than others to the pandemic's negative health, economic, and social consequences (Bottan et al., 2020; Cuéllar et al., 2021; Nanda, 2020; Onyango et al., 2021). In their pandemic impact typology, Katikireddi et al. (2021) propose that these inequalities produced impact ‘pathways’ which include initial exposure to the coronavirus, vulnerability to infection/disease, its social and economic consequences, effectiveness of pandemic control measures, and the adverse consequences of control measures. Along all these pathways, migrants working in other countries or away from home in their own countries have proven to be the most vulnerable and negatively affected (Abu Alrob & Shields, 2022; Freier & Vera Espinoza, 2021; Jesline et al., 2021; Mengesha et al., 2022; Mukumbang et al., 2020; Quandt et al., 2021; Ramachandran et al., 2022).

For this paper, we hypothesized that the prepandemic conditions and vulnerabilities of migrants generated pathways which led to greater food insecurity for migrant households and different food security outcomes to nonmigrants. For our theoretical framing, we adopted the standard FAO definition of food security as pertaining when ‘all people, at all times, have physical and economic access to sufficient, safe and nutritious food that meets their dietary needs and food preferences for an active and healthy life’ (World Food Summit, 1996). Globally, the COVID‐19 crisis has reduced the physical and economic access of millions of migrants to sufficient, safe and nutritious food, compromised their food needs and preferences, and subverted their ability to pursue active and healthy lives (Smith & Wesselbaum, 2020).

There is a general consensus in the food security literature that the standard definition has four essential elements which we have adjusted to foreground the experience of migrant populations (Leroy et al., 2015):

- Availability: there is a reliable and consistent supply of good quality food for a balanced diet for all migrants;
- Accessibility: migrants have the resources to ensure physical and economic access to a healthy food environment;
- Utilization: migrants are able to prepare and consume nutritious, culturally‐appropriate, preferred and safe foods;
- Stability: both the quantity and quality of food available and accessible to migrants remain stable over time and are not reduced by shocks and crises.

**Figure 1:**
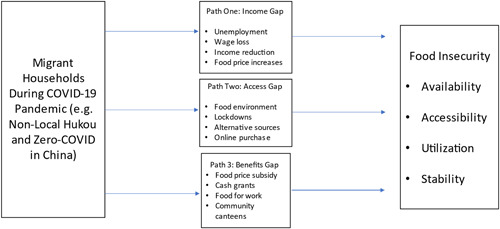
Pathways to Migrant Food Insecurity During COVID‐19

In Figure 1, we suggest that there are three main pathways to increased food insecurity for migrants during COVID‐19: (i) an income pathway involving insufficient income for food purchase; (ii) a food access pathway involving poor or limited access to food outlets; and (iii) a social benefit pathway involving the absence or denial of social assistance for migrants.

#### 2.3.1 Income pathway

In circumstances of adequate food availability, level of income is the primary determinant of household food security (Babatunde & Qaim, 2010; Bashir & Schilizzi, 2013; Owusu et al., 2011). As a rule, food security improves with income even as the proportion of total income spent on food declines. In China, the level of individual and household income is therefore likely to be a key factor in the food security outcomes of households who do not have local hukou in cities. Many urban migrants were previously farmers with low levels of education and vocational skills. In the cities, migrants engage in low‐skilled and physically demanding jobs with low entry barriers (K. Chan & Yang, 2020; Liu et al., 2020; Tianhong et al., 2000). Compared with other, more stable, occupations, the jobs undertaken by migrants are largely temporary and low‐waged (Gu et al., 2020). The lower incomes of migrant households mean access to fewer affordable food options and a lower frequency of consuming nutritious food. As migrants are usually engaged in low‐income jobs, they are more likely to be affected by the shutdown of economic activity during the COVID‐19 pandemic. While income loss increases migrant vulnerability to food insecurity, so does rising food prices which make preferred foods less affordable with a reduction in dietary diversity. Combined, loss of income and rising food prices create a ‘hukou‐income gap‐food security’ path.

#### 2.3.2 Food accessibility pathway

Turner et al. (2018) define the urban food environment as ‘the spaces within which food acquisition occurs, and the series of market‐based opportunities and constraints that influence people's food acquisition and consumption’. In China, the central and city governments have prioritised comprehensive food environment planning that maximizes the physical and economic access of all urban residents to food markets including wet markets, supermarkets, and wholesale markets (Zhong et al., 2019, Zhong, Si, et al., 2021). As a result, the neighbourhood food environment may not be all that different for migrants and nonmigrants although newer residential developments and higher‐income areas tend to be better served. However, familiarity with the broader city food environment may vary especially as it can take time to get to know. While migrants can probably identify the food outlets in and around the areas where they shop, they probably have less knowledge of food outlets further away from where they live. In the normal course of events, this may not particularly matter for migrants. However, when their familiar food sources are abruptly closed or the quality and availability of food sold is reduced, as during the pandemic lockdown, they encounter additional difficulties accessing food and experience heightened food insecurity.

Migrants may also have limited access to newer food retail sources such as neighbourhood‐based group buying, which is commonly organized online through WeChat or other social media apps (Dai & Qi, 2020). Neighbourhood‐based buying groups tend to be more common amongst residents with local hukou. Additionally, migrant households do not have as strong social networks as those with local hukou, as they have fewer relatives and friends in the city. Social networks are important for accessing food and information about food, so migrants do not enjoy the same access to social network‐supported food supplies. In emergencies, local households can turn to relatives and friends for help and share food channels through their networks, unlike migrants. All these relative disadvantages create a ‘hukou‐food market access gap‐food security’ path with decreased food accessibility and increased food insecurity among migrants.

#### 2.3.3 Social security pathway

Social protection programmes are an important mechanism for mitigating chronic food insecurity in normal times and averting hunger at times of crisis (Devereux, 2016, 2021). In Chinese cities, residents with local hukou have access to a range of social benefits which are not available to migrants (K. W. Chan & Zhang, 1999; Gu et al., 2020). For example, households with nonlocal hukou do not enjoy the same access to children's education, healthcare, and state‐subsidized benefits (Afridi et al., 2015; Hung, 2022; Kuang & Liu, 2012; Niu & Qi, 2015; Song & Smith, 2019; Song & Zhou, 2019; X. Wang et al., 2017; Wu & Wang, 2014; Zhan, 2011). They also do not have the same access in urban areas to social protection programmes such as child grants, old age grants, and minimum living standards allowances. They are more likely to be able to access food for work programmes only where hukou status is not an issue.

Many cities in China also require that home buyers have a local hukou to purchase a property. When urban households buy an apartment, ownership is recorded by a property management company which runs the residential complex. Households without local hukou in the city are more likely to be tenants and are *de facto* excluded from registration by property management companies. During the early part of 2020 and subsequent lockdowns, many property management companies organized emergency food group buying services for residents with recorded ownership (Zhong, Crush, et al., 2021). These difference in benefits and services by hukou make migrant households more vulnerable to food security, a path we refer to as hukou‐social benefit gap‐food security’.

In the remainder of the paper, we draw on the theoretical framing of pathways to food insecurity to illuminate a case study of the city of Nanjing, China. The paper addresses three basic questions about the impact of COVID‐19 on migrant food security in the city:

(a) Did migrant households without local hukou in Nanjing experience food insecurity in the initial phase of the pandemic and, if so, what forms did this take?
(b) Were migrant households without local hukou in Nanjing more likely than those with local hukou to experience food insecurity?
(c) Which pathways to migrant food insecurity discussed in this section help to explain the food security experience of migrants in Nanjing during the pandemic?

## 3 STUDY METHODOLOGY

### 3.1 Study site

Nanjing is the capital city of Jiangsu Province, located in eastern China, about 300 kilometres west of Shanghai. The population of the city increased by in‐migration and natural population growth from 8.01 million in 2010 to 9.32 million in 2020 (Nanjing Statistical Yearbook, 2021). In Jiangsu Province, rural unemployment is a major driver of migration to cities such as Nanjing (Lyu et al., 2019). Nanjing was selected as the study site for three main reasons. First, the city has a sizeable migrant population. The number of nonlocals increased from 1.91 million in 2010 to 2.65 million in 2020 accounting for 28.5% of the urban population in 2020 (Nanjing Municipal Bureau of Statistics, 2021). Second, there is a considerable body of prior research on the food system of Nanjing, included a pre‐pandemic household food security survey which provides a baseline from which to measure the impact of the COVID‐19 pandemic on the food security of households (Si & Zhong, 2018). Finally, Nanjing was selected as the research site because its pandemic experience is typical of many second‐tier cities in the country. Measures taken by the local authorities to contain the spread of COVID‐19 in these centres included mobility controls, complete or partial lockdown of residential communities, and encouragement of online food purchasing and delivery. While residents were required to stay at home and had restrictions on their everyday mobility, supermarkets and wet markets generally remained open to offer daily necessities and the public transportation system was still in operation

### 3.2 Household survey

This paper uses data from an online survey of household food security conducted in March 2020 in Nanjing. Mobility restrictions and lockdown measures in early 2020 made it impossible to randomly sample local and nonlocal households in the city and implement a face‐to‐face survey. The online survey was therefore designed and implemented using the electronic questionnaire platform Wenjuanxing. The platform ensured that only residents of Nanjing could access the survey by limiting the IP addresses to those in the city. WeChat was used to distribute the questionnaire. The sampling strategy was an online variant of ‘snowball sampling’, a non‐probabilistic sampling method to locate respondents through other respondents especially useful for identifying specific target groups (Etikan & Bala, 2017; Etikan et al., 2016).

The survey targeted all residents of Nanjing to increase the number of respondents and to potentially allow for comparisons between those with and without local hukou. In the final analysis, a total of 1445 responses were received from Nanjing residents and after screening for incomplete surveys, there were 1199 validated questionnaires. Those responses with vacant values for the variables used in this analysis were removed from the data set, leaving a total of 536 validated questionnaires. Household migration status was set as the control variable in the analysis. To distinguish between migrant and urban households, household registration status was used. Households with Nanjing hukou were classified as local or nonmigrant. Households whose hukou was not in Nanjing were classified as migrant. A total of 431 surveyed households (80%) had Nanjing hukou and 105 households (20%) were migrants with non‐Nanjing hukou.

Four main types of information were collected from respondents. First, basic information about the household, including size, membership, structure, housing type, property rights, and hukou status was collected. Second, respondents were asked what kinds of lockdown (complete or partial) and quarantine measures their residential community had experienced. Third, the survey collected detailed information about household food purchasing and consumption in the previous month. Finally, to assess the extent of food security, all households were asked nine frequency‐of‐occurrence questions derived from Coates et al. (2007) and designed to capture different dimensions of household food insecurity.

### 3.3 Food security metrics

The nine frequency‐of‐occurrence questions capture the overall level of household food insecurity in Nanjing during the COVID‐19 pandemic (Table 1). These questions form the basis of two standardized and validated cross‐cultural food security metrics: the Household Food Insecurity Access Scale and the Household Hunger Scale (Ballard et al., 2011; Coates et al., 2007; Leroy et al., 2015) Three additional measures were used to identify the different dimensions of food insecurity: Food Anxiety, Limited Food Quality, and Insufficient Food Quantity (Table 2). The coding of the five food security measures used as dependent variables in the analysis was as follows:

- Household Food Insecurity Access Scale (HFIAS) (Q1‐9) is an overall measure of food insecurity based on all nine questions in Table 1. The frequency‐of‐occurrence for each question coded as 0 (Never), 1 (Rarely), 2 (Sometimes) and 3 (Often). The scale allocates each household a score ranging from 0 to 27. The higher the HFIAS score, the more food insecure the household, and the smaller the score, the more food secure the household.
- Household Hunger Scale (HHS) (Q7‐9) is a secondary indicator focused on household responses to food shortages and hunger. The scale assigns the following values: 0 (Never), 1 (Rarely/Sometimes) and 2 (Often). The range of HHS scores is therefore 0–6. The higher the HHS score, the more intense the household experience of hunger, the lower the score, the less the experience of household hunger.
- Food Anxiety (FA) (Q1) captures the frequency of uncertainty or anxiety about the household food supply. This metric is an ordinal four‐category variable: never, rarely, sometimes and always.
- Limited Food Quality (LFQ) (Q2‐4) captures the quality and diversity of the household diet on a scale ranging from 0 (good quality and diversity) to 9 (poor quality and diversity).
- Insufficient Food Quantity (IFQ) (Q5‐9) is a measure of whether the quantity of food to which the household has access is sufficient to meet household needs on a scale from 0 (completely sufficient) to 15 (extremely insufficient).

**Table 1:** Food security questions

In the past 4 weeks:
Q1: Did you worry that your household would not have enough food?
Q2: Were you or any household member not able to eat the kinds of foods you preferred because of a lack of resources?
Q3: Did you or any household member have to eat a limited variety of foods due to a lack of resources?
Q4: Did you or any household member have to eat some foods that you really did not want to eat because of a lack of resources to obtain other types of food?
Q5: Did you or any household member have to eat a smaller meal than you felt you needed because there was not enough food?
Q6: Did you or any household member have to eat fewer meals in a day because there was not enough food?
Q7: Was there ever no food to eat of any kind in your household because of lack of resources to get food?
Q8: Did you or any household member go to sleep at night hungry because there was not enough food?
Q9: Did you or any household member go a whole day and night without eating anything because there was not enough food?

*Source*: Coates et al. (2007).

**Table 2:** Dependent and independent variables

	Definitions and coding
*Dependent variables food security status*	
HFIAS (HFIAS score)	Household food insecurity access scale score, ranging from 0 to 27
HHS (HHS score)	Household hunger scale score, ranging from 0 to 6
FA (food anxiety)	Household food anxiety level, ranging from 0 to 3
Never	
Rarely	
Sometimes	
Often	
LFQ (limited food quality)	Limited food diversity and unsatisfied food preferences, ranging from 0 to 9
IFQ (insufficient food quantity)	Insufficient food for consumption in households, ranging from 0 to 15
*Independent variablesmigration status (explanatory)*	
*Hukou*	If the household has a Nanjing hukou, *hukou* = 1; otherwise, *hukou* = 0
Local households	
Migrant households	
*Household characteristics (control)*	
NFT (number of food types)	Number of types that were affected in household food consumption (0–22)
HT (household type)	If the household is female‐headed (without a male partner), HT = 1; otherwise, HT = 0
Female‐centred households	
Other households	
FE (food expenditure)	If household spent more money on food than before COVID‐19 pandemic, FE = 1; otherwise, FT = *0*
Higher than prepandemic	
Equal to/lower than prepandemic	
HS (household size)	If the household members are less than 5, Household size = 1; otherwise, household size = 0
Five members or less	
More than five members	
ME (medical expenditure)	Household medical expenses because of COVID‐19 (CNY)

### 3.4 Household characteristics

Five variables reflecting different household characteristics during the pandemic were included in the analysis (Table 2): (i) Number of common food types (NFT) foregone captured by the question ‘Has the COVID‐19 outbreak affected your consumption of the following foods?’ Respondents were presented with a list of 24 common food types to respond to; (ii) household type (HT)—female‐centred (i.e., households with a female head and no male spouse/partner) and other; (iii) food expenditure (FE) more than before COVID‐19 or the same/less than before; (iv) household size (HS) of less or more than five members and (v) COVID‐related medical expenditure (ME).

### 3.5 Data analysis and limitations

Five multiple regression models were used to compare the food security of migrant and nonmigrant households and determine the significance of any differences between them. Model I used the HFIAS as the dependent variable to represent the overall experience of household food insecurity during the pandemic. Model II used the HHS as the dependent variable to represent the frequency of experience of hunger during the pandemic. Model III used IFQ as the dependent variable to represent the frequency of insufficient food intake. Model IV used LFQ as the dependent variable to represent the frequency of consuming undesirable foods.

The analysis and conclusions do have several limitations. First, pandemic restrictions meant that the household sample was not randomly selected and the results are therefore not necessarily representative of the population as a whole. However, they do provide important insights and provisional explanations for migrant food insecurity during COVID‐19. Second, the relatively small sample of migrant households reflects the difficulties of accessing the ‘floating population’ through online surveys. In particular, the methodology may have under‐sampled households living in lower‐income areas of the city. Third, the distinction between migrant and nonmigrant households by hukou means that migrant households that have acquired Nanjing hukou are not considered as part of the floating population of the city. Finally, by focusing on the household as a unit of data collection and comparison, the individual experience of household members and intra‐household dynamics is not captured in the analysis.

## 4 RESULTS

### 4.1 Food access

This section of the paper compares migrant and nonmigrant households in Nanjing during the early weeks of the pandemic using descriptive statistics. Table 3 compares the major food‐related challenges identified by the two groups of respondents. Migrants had greater restrictions on their mobility, more restricted access to wet markets and supermarkets, and higher loss of income. Other challenges affected both groups more equally. Table 4 shows that migrants were able to access alternative food sources in roughly similar numbers as local households. For example, 53% of locals accessed online buying groups, but so did 51% of migrants. Finally, Table 5 shows the main foods whose consumption was negatively affected by the pandemic, most of which are staples in the Chinese diet.

**Table 3:** Major challenges to food access

	% of migrant households	% of local households
Increased expenditure on food	79.0	64.7
Restricted mobility	39.0	27.8
Restricted access to wet markets and supermarkets	36.2	30.2
Food price increases	37.1	35.5
Loss of income	25.7	19.3
Limited food availability/variety in wet markets and supermarkets	26.7	26.5
Limited food availability/variety at online stores	12.4	19.3
Food quickly sells out in online stores	14.3	15.1
Restricted food delivery to home	13.3	9.3

**Table 4:** Patronage of alternative food sources

	% of migrant households	% of local households
Online buying groups	50.5	52.7
Property management committees	16.2	11.6
Neighbourhood committees	9.5	5.8
Volunteers	6.7	7.4

**Table 5:** Food types most negatively affected by pandemic

	% of migrant households	% of local households
Pork	35.2	20.2
Beef and lamb	27.6	14.8
Leafy greens	24.8	20.9
Fruits	21.0	10.9
Fish	20.0	18.8
Tofu, bean curds, other foods made from soybeans	15.2	6.7
Poultry	14.3	15.1
Tubers	14.3	7.7

### 4.2 Types of household food insecurity

Table 6 provides an overview of the findings from all respondent households combined. The average HFIAS score was 4.82 out of a possible 27 (standard deviation [SD] = 5.51) which represents a significant increase from an earlier prepandemic survey in Nanjing when the HFIAS was only 0.61 (Si & Zhong, 2018). Just over 40% of households had never worried that the household would not have enough food, while 30% had sometimes or often worried. The mean Household Hunger Score was 0.5 out of a possible 6. As an HHS of 0–1 indicates that there is little hunger in a household, this suggests that an inadequate quantity of food was not a major issue for the average household. The mean IFQ and LFQ were 1.42 and 2.42, respectively, suggesting that food quality was a more important challenge for households than the amount of food they could access. The number of food types that households had gone without as a direct result of the pandemic was 2.29 on average (SD = 3.48).

**Table 6:** Mean household values

Variables	%	Mean	SD
*Food security status (dependent)*			
HFIAS (HFIAS score)		4.82	5.21
HHS (HHS score)		0.50	1.17
FA (food anxiety)			
Never	40.6		
Rarely	28.7		
Sometimes	22.4		
Often	8.2		
LFQ (limited food quality)		2.42	2.43
IFQ (insufficient food quantity)		1.42	2.92
*Migration status (explanatory)*			
*Hukou*			
Local households	80.4		
Migrant households	19.6		
*Household characteristics (control)*			
NFT (number of food types)		2.29	3.48
HT (household type)			
Female‐centred households	10.6		
Other households	89.4		
FE (food expenditure)			
Higher than prepandemic	65.5		
Equal to/lower than prepandemic	34.5		
HS (household size)			
Five members or less	93.1		
More than five members	6.9		
ME (medical expenditure)		1.07	1.72

### 4.3 Migrant and local food insecurities

The analysis reveals several differences in levels of food insecurity between local households with Nanjing hukou and migrant households without Nanjing hukou (Table 7). On the various food security metrics, migrant households performed worse than local households. Migrant households had higher levels of anxiety about their food supply (migrant: 38%; local 29%) and scored an average 6.86 on the HFIAS, compared with 4.33 for local households. Similarly, the HHS (migrant: 0.89; local 0.44) and IFQ (migrant: 2.46; local 1.46) were both higher for migrant households, suggesting that they experienced greater challenges with accessing enough food. In addition, the index of LFQ was higher for migrants (migrant: 3.15; local: 2.24). On average, migrant households therefore experienced greater anxiety, more hunger, reduced food quantity and more constraints on food quality. The number of food types gone without was also higher for migrants (migrant: 3.15; local: 2.08).

**Table 7:** Food insecurity of local and migrant households

	All households	Local households	Migrant households
Sample size	536	431	105
Percentage	100%	19.59%	80.41%
*Variable classification*	*Mean (standard deviation)/number (%)*		
HFIAS (HFIAS score)	4.82 (5.51)	4.33 (4.98)	6.86 (6.94)
HHS (HHS score)	0.50 (1.17)	0.40 (1.04)	0.89 (1.56)
IFQ (insufficient food quantity)	1.42 (2.92)	1.16 (2.55)	2.46 (3.95)
LFQ (limited food quality)	2.42 (2.43)	2.24 (2.32)	3.15 (2.73)
FA (food anxiety)			
Never	218 (40.67)	186 (43.16)	32 (30.48)
Rarely	154 (28.73)	121 (28.07)	33 (31.43)
Sometimes	120 (22.39)	98 (22.74)	22 (20.95)
Often	44 (8.21)	26 (6.03)	18 (17.14)
NFT (number of food types)	2.29(3.48)	2.08(3.39)	3.15(3.75)
FE (food expenditure)			
Higher than prepandemic	351(65.49)	271(62.88)	80(76.19)
Equal to/lower than prepandemic	185(34.51)	160(37.12)	25(23.81)

### 4.4 Modelling food insecurity

Table 8 shows the results of the statistical regression analysis of the survey data. We generated five models with the different food security indicators as the dependent variables: Model I (HFIAS), Model Ⅱ (HHS), Model Ⅲ (IFQ), Model Ⅳ (LFQ) and Model Ⅴ (FA). Model I shows that as the number of food types affected by the COVID‐19 pandemic increased, so did household food insecurity. Female‐centred households and those with higher expenditures on food were also more likely to be food insecure. Larger households and households that spent more on medical needs were less likely to be food insecure.

**Table 8:** Regression results for the impact of hukou on household food insecurity

Dependent variables/independent variables	Model Ⅰ	Model Ⅱ	Model Ⅲ	Model Ⅳ	Model Ⅴ
	HFIAS	HHS	IFQ	LFQ	FA
*Hukou*	−1.277**	−0.251*	−0.678*	−0.423	−0.344
	(0.624)	(0.141)	(0.353)	(0.269)	(0.212)
Number of food types (NFT)	0.612***	0.111***	0.289***	0.238***	0.172***
	(0.101)	(0.024)	(0.059)	(0.041)	(0.029)
Household type (HT)	1.606**	0.294	0.835*	0.704**	0.194
	(0.725)	(0.180)	(0.448)	(0.296)	(0.249)
Food expense (FE)	1.157***	0.188**	0.585***	0.401**	0.323*
	(0.401)	(0.089)	(0.210)	(0.195)	(0.172)
Household size (HS)	2.057***	0.075	0.482	1.011***	1.438***
	(0.681)	(0.176)	(0.404)	(0.302)	(0.437)
Medical expense (ME)	0.460**	0.089**	0.222*	0.175***	0.157**
	(0.188)	(0.044)	(0.114)	(0.060)	(0.078)
Constant	1.113	0.126	0.146	0.751*	/
	(0.885)	(0.218)	(0.521)	(0.388)	/
*N*	536	536	536	536	536
R‐squared	0.247	0.174	0.196	0.192	/
Pseudo *R*2	/	/	/	/	0.070
Regression approach	Ordinary least squares				Ordered logistic regression

*Note*: The values in parentheses are robust standard errors. *, **and *** denote significance at 10%‐level, 5%‐level, 1%‐level.

The first three models all confirm that migrant status (the independent variable hukou) had a statistically significant impact on food security. Model 1 indicates that migrant status had a significant negative impact on the overall food security (HFIAS) score. The value of the coefficient is −1.277 which means that the HFIAS of households with Nanjing hukou was 1.277 times lower than that of households without Nanjing hukou, holding other variables constant. Models II and III use household hunger and insufficient food quantity respectively as the dependent variable. Both models indicate that migrant households were more likely to be affected by hunger and food shortages than local households, holding other variables constant. Model IV shows no significant difference in the quality of food consumed between local and migrant households during the pandemic. Model V (FA) indicates that there was no significant difference in anxiety about the food supply between local and migrant households.

## 5 DISCUSSION

The analysis in the previous section suggests that the hukou system was an important determinant of household food security outcomes in Nanjing during the first wave of the COVID‐19 pandemic. Across a variety of metrics, households without local Nanjing hukou had more negative outcomes than those with Nanjing hukou. The latter certainly felt the impact of the pandemic and experienced an overall decline in food security but the decline was not as severe overall as it was for migrants. Disaggregating the different dimensions of food insecurity, migrant households on average experienced greater anxiety, greater difficulty in accessing enough food, and a greater deterioration in the quality of food consumed. The regression analysis suggests difficulty in accessing enough food was the most significant difference.

With reference to the theoretical framework for conceptualizing migrant food insecurity in Nanjing during the COVID‐19 pandemic, food continued to come into the city from the countryside without major shortages as the city's food supply chains were relatively resilient especially after the government introduced measures to minimize disruption to the food supply. The analysis indicates that migrant households experienced greater problems with the accessibility and utilization dimensions of the standard definition. Given the short recall period of the pandemic itself, conclusions about the stability of household food consumption over time were not possible. Future research would need to examine the migrant experience of the three years of the pandemic to assess whether food security recovered after the first wave and whether there were further bouts of insecurity as the local economy continued to be affected by the pandemic.

Figure 1 identified three potential pathways to increased food insecurity for migrant households during the pandemic. The ‘hukou‐income gap‐food security’ path asks if migrants experienced a loss of income, whether they were affected by food price increases, and whether these changes impacted their ability to purchase food of sufficient quality and quantity. One survey respondent replied to all these questions in the affirmative: ‘There is no source of income, it is expensive to buy food, and I almost have no money to make a living’ (Respondent No. 151). A minority of migrants (around 26%, slightly higher than the 19% of those with local hukou) reported lost income; but as many as three‐quarters said that food was more expensive than before the pandemic. This suggests that increased cost rather than income loss was more important for most migrant households.

The second pathway (hukou‐food market access gap‐food security) addresses the issue of food access during the pandemic. In the early weeks, restaurants, shopping malls, schools, and other places were closed to prevent the spread of the virus, However, unlike in Wuhan, food outlets such as wet markets and supermarkets remained open. Although normal access to these important food sources was reduced by residential lockdowns and controls on personal mobility, as many as 96% of migrant households in Nanjing reported that they had bought food from physical stores in the previous month. While local households were able to gain access to alternative emergency food sources, this was potentially more difficult for migrants. These sources included online buying clubs, and food purchasing and distribution by residential property management committees, local government neighbourhood committees. and volunteers. Around 44% of migrant respondents had never utilized any of these methods of procuring food. However, there was no significant difference between migrants and locals in the usage of each method. Online food purchasing grew dramatically during the pandemic, and it is a mark of the pervasive use of IT that 76% of migrants had bought food online. The primary complaints of households that used these alternative sources was that they were expensive, that the produce was insufficiently fresh, and that they had to buy multi‐item food packages rather than individual products.

The third pathway (hukou‐benefit gap‐food security) asks whether households were able to access social benefits to mitigate food insecurity. At the time of the survey, it was not clear what these emergency benefits were and who could access them. Subsequently it emerged that by the end of July 2020, the Nanjing government had granted a total of 89 million RMB of ‘temporary price subsidies’ benefiting 652,800 residents. However, subsidies are distributed monthly in the place where hukou is registered, effectively precluding access by migrant households who would otherwise have qualified.

Of the three possible pathways to food insecurity by migrant households, the income‐gap path was the most important in Nanjing. After an initial shock from a short complete lockdown, the potential food market access path was not a major challenge for most migrant households, unlike in Wuhan with its extended hard lockdown. Wet markets and supermarkets remained open in Nanjing (albeit with pandemic precautions such as strict social distancing) and many migrants had access to online food purchasing. The analysis in this paper suggests that of the various components of food insecurity, the decline in food quality was more important overall than the loss of food quantity. Migrant households experienced the greatest challenges in accessing pork, beef, fish, leafy greens, and fruits. We can infer that this was largely because of price increases that made these foods unaffordable rather than being a result of income loss. At the same time, a minority of migrant households did experience income loss and food security challenges relating to food quantity as well as quality. These are also the households without access to emergency pandemic measures such as alternative sources of food supply and online purchasing. Without access to the temporary price subsidy programme, these households were affected negatively by the benefit gap.

## 6 CONCLUSION

The paper is a contribution to understanding China's early pandemic experience for migrants but also offers some pointers of broader relevance. First, negative COVID‐19 food security outcomes cannot be separated from the operation of the hukou system at the city scale. Internal migrants elsewhere may not face the same regulatory constraints but low‐income, temporary workers in precarious employment are just as likely to experience adverse food security outcomes irrespective of place. Second, the paper suggests that the impact of COVID‐19 containment on food security outcomes is likely to be more severe for migrants than non‐migrants. While pandemic control and mitigation measures by central and local government in China disrupted urban food systems and led to a generalized increase in food insecurity, migrant households were especially vulnerable and had worse food security outcomes than non‐migrants. This is likely to have been the case within other countries as well. Third, this study suggests that in China, a decline in dietary quality and the nutritional value of food consumed was more important than absolute food shortages for most migrant households. While this may not be as true elsewhere, it is important everywhere to use indicators that capture the different dimensions of food insecurity. Finally, this paper implies that migrants should not be treated as a homogenous group. In their small‐scale study of migrants in Nanjing during COVID‐19, Tang and Li (2021) suggest that it is important to appreciate differences within the migrant population in terms of access to stable employment, shelter, housing, and family and kinship support in the city and the countryside. The same applies to the experience of food insecurity as the food security impacts of COVID‐19 were not equally felt by all migrants.

In the context of the issues raised in the Introduction, Orjuela‐Grimm et al. (2022) issue a call for a new interdisciplinary research agenda to document the food insecurity dynamics and experiences of migrants on the move. In the context of the pandemic, Oliva‐Arocas et al. (2022) note that migrants are ‘a group specifically affected but poorly studied’. There has been a particular dearth of analysis on the impacts of COVID‐19 on the food security of migrant populations. In the introduction to the paper, we noted the main contributions that the paper aims to make to the literature on the migration and food security nexus in pandemic times. Here we revisit these issues with recommendations for future research priorities. There are over 750 million internal migrants around the globe, all of whom were impacted to some degree by the pandemic. Surveys like this in other regions would help confirm whether other countries and cities, and migrants themselves, were better able to navigate the food security challenges of the pandemic.

The food security impacts of the pandemic on migrants are very likely to differ across space and from place to place. Even within China, the experience and food security outcomes of the pandemic varied considerably between neighbouring Nanjing and Wuhan (Zhong, Crush, et al., 2021). This suggests that further case studies from around the globe would be invaluable in nuancing meta‐narratives about the impact of COVID‐19 on migrant food security. One of the key unanswered questions in most countries is whether the pandemic disruptions of early 2020 were enduring or temporary. Have pre‐pandemic levels of food security been restored or do migrant households still feel its effects two years later? This is of particular importance in building resilience to better cope with the food insecurity consequences of future waves of COVID‐19 or other pandemics. Finally, the theoretical framework which guided this study highlights the role of various potential pathways to food insecurity confronting migrants during the pandemic and, as such, should be of utility to future studies of migrant food insecurity during COVID‐19 in China and elsewhere.

## CONFLICT OF INTEREST

The authors declare no conflict of interest.

## Data Availability

The data that support the findings of this study are available from the corresponding author upon reasonable request.
